# The modulation of the hexosamine biosynthetic pathway impacts the localization of CD36 in macrophages

**DOI:** 10.3389/abp.2024.13004

**Published:** 2024-07-08

**Authors:** Karen Julissa Loaeza-Reyes, Edgar Zenteno, Eleazar Ramírez-Hernández, Roberta Salinas-Marin, Adriana Moreno-Rodríguez, Rafael Torres-Rosas, Liliana Argueta-Figueroa, Berenice Fernández-Rojas, Socorro Pina-Canseco, Alfonso E. Acevedo-Mascarúa, Alicia Hernández-Antonio, Yobana Pérez-Cervera

**Affiliations:** ^1^ Centro de Estudios en Ciencias de la Salud y la Enfermedad, Facultad de Odontología, Universidad Autónoma Benito Juárez de Oaxaca, Oaxaca, Mexico; ^2^ Centro de Investigación Multidisciplinaria Facultad de Medicina-UNAM-UABJO, Universidad Autónoma Benito Juárez de Oaxaca, Oaxaca, Mexico; ^3^ Departamento de Bioquímica, Facultad de Medicina, Universidad Nacional Autónoma de México, Mexico City, Mexico; ^4^ Laboratorio de Glicobiología Humana y Diagnóstico Molecular, Centro de Investigación en Dinámica Celular, Instituto de Investigación en Ciencias Básicas y Aplicadas, Universidad Autónoma del Estado de Morelos, Cuernavaca, Mexico; ^5^ Facultad de Ciencias Químicas, Universidad Autónoma Benito Juárez de Oaxaca, Oaxaca, Mexico; ^6^ CONAHCYT – Facultad de Odontología, Universidad Autónoma Benito Juárez de Oaxaca, Oaxaca, Mexico

**Keywords:** CD36 localization, O-GlcNAcylation, hexosamine biosynthetic pathway, vesicular traffic, environment of cells

## Abstract

CD36 is a type 2 cell surface scavenger receptor expressed in various tissues. In macrophages, CD36 recognizes oxidized low-density lipoprotein (ox-LDL), which promotes the formation of foam cells, the first step toward an atherosclerotic arterial lesion. CD36 possesses a variety of posttranslational modifications, among them N-glycosylation and O-GlcNAc modification. Some of the roles of these modifications on CD36 are known, such as N-linked glycosylation, which provides proper folding and trafficking to the plasma membrane in the human embryonic kidney. This study aimed to determine whether variations in the availability of UDP-GlcNAc could impact Rab-5-mediated endocytic trafficking and, therefore, the cellular localization of CD36. These preliminary results suggest that the availability of the substrate UDP-GlcNAc, modulated in response to treatment with Thiamet G (TMG), OSMI-1 (O-GlcNAcylation enzymes modulators) or Azaserine (HBP modulator), influences the localization of CD36 in J774 macrophages, and the endocytic trafficking as evidenced by the regulatory protein Rab-5, between the plasma membrane and the cytoplasm.

## Introduction

Human CD36 is a class B scavenger receptor localized to the plasma membrane that is widely expressed in immune cells like macrophages, monocytes, dendritic cells and subsets of T and B cells, and non-immune cells, such as platelets, immature erythrocytes adipocytes, myocytes, specialized epithelial cells, and microvascular endothelial cells. Its ligands include endogenously derived ligands such as apoptotic cells, cholesterol esters, fatty acids, oxidatively modified lipoproteins, glycated proteins, and amyloid-forming peptides (Luiken et al., 2016; Chen et al., 2022). Similarly, CD36 is a pattern recognition receptor for molecular patterns presented by various pathogens on phagocytic cells, binds, and mediates phagocytosis (Patel et al., 2004; Baranova et al., 2008; Silverstein and Febbraio, 2009).

The endocytic system of eukaryotic cells embraces a complex network of membrane compartments, each of which fulfills a specific set of tasks in cargo sorting, distribution, and catabolism (Barbero et al., 2002). Rab GTPases organize the endocytic pathway into a mosaic of biochemically and functionally distinct membrane domains; they are involved in vesicle trafficking and endocytic and exocytic processes (Rink et al., 2005). Rab5 recruits different effector proteins that oligomerize within a membrane domain enriched in PI(3)P (Christoforidis et al., 1999). Rab5, Rab4, and Rab11 occupy distinct membrane domains that are sequentially traversed by recycling cargo. Cargo destined for degradation is first internalized into Rab5 domains in early endosomes (Yuan and Song, 2020) and later appears in Rab7 domains in late endosomes (Mukhopadhyay et al., 1997).

Short-term (minutes) regulation of cellular fatty acid uptake rate occurs by reversible intracellular recycling (vesicular traffic) of CD36 from an endosomal storage compartment to the plasma membrane. Long-term regulation of cellular fatty acid uptake occurs via changes in CD36 gene transcription, mediated, among others, by fatty acid-induced PPAR activation, HIF-1, and C/EBPα (Glatz and Luiken, 2018). CD36 expression is regulated at both the transcriptional and posttranslational levels; the regulation differs between different cell types. Post-translational modifications (PTMs) modulate partner-to-partner interactions and are responsible for protein function. A single PTM or a combination of PTMs modifies the local conformation of a region or domains of a protein and offers different interaction surfaces. Therefore, a protein can interact with many partners, changing its localization, stability, interaction with other partners, and, ultimately, its activities (Vercoutter-Edouart et al., 2015). The evidence concerning PTMs of CD36 suggests that these modifications impact protein function in a tissue-specific way; for more details, see the reviews (Shu et al., 2022; J.J; Luiken et al., 2016).

In addition, CD36 is heavily N-linked glycosylated (Mori et al., 2012), which is the cause for the increase in molecular mass to 88 kDa. N-glycosylation of membrane and secretory proteins impacts proper folding and trafficking to the plasma membrane and enhances their stability (Hoosdally et al., 2009). N-glycosylation occurs in the endoplasmic reticulum (ER) and refers to the attachment of the oligosaccharide N-acetylglucosamine (GlcNAc) to the side chain of Asparagine (Asn) through a β-1N linkage of a protein molecule—N-glycans impact the structure and function of some eukaryotic proteins (Reily et al., 2019). The endoplasmic reticulum and the Golgi apparatus synthesize glycolipids, glycoproteins, and glycans utilizing UDP-GlcNAc, which is synthesized through the Hexosamine Biosynthetic Pathway (HBP).

**FIGURE 1:**
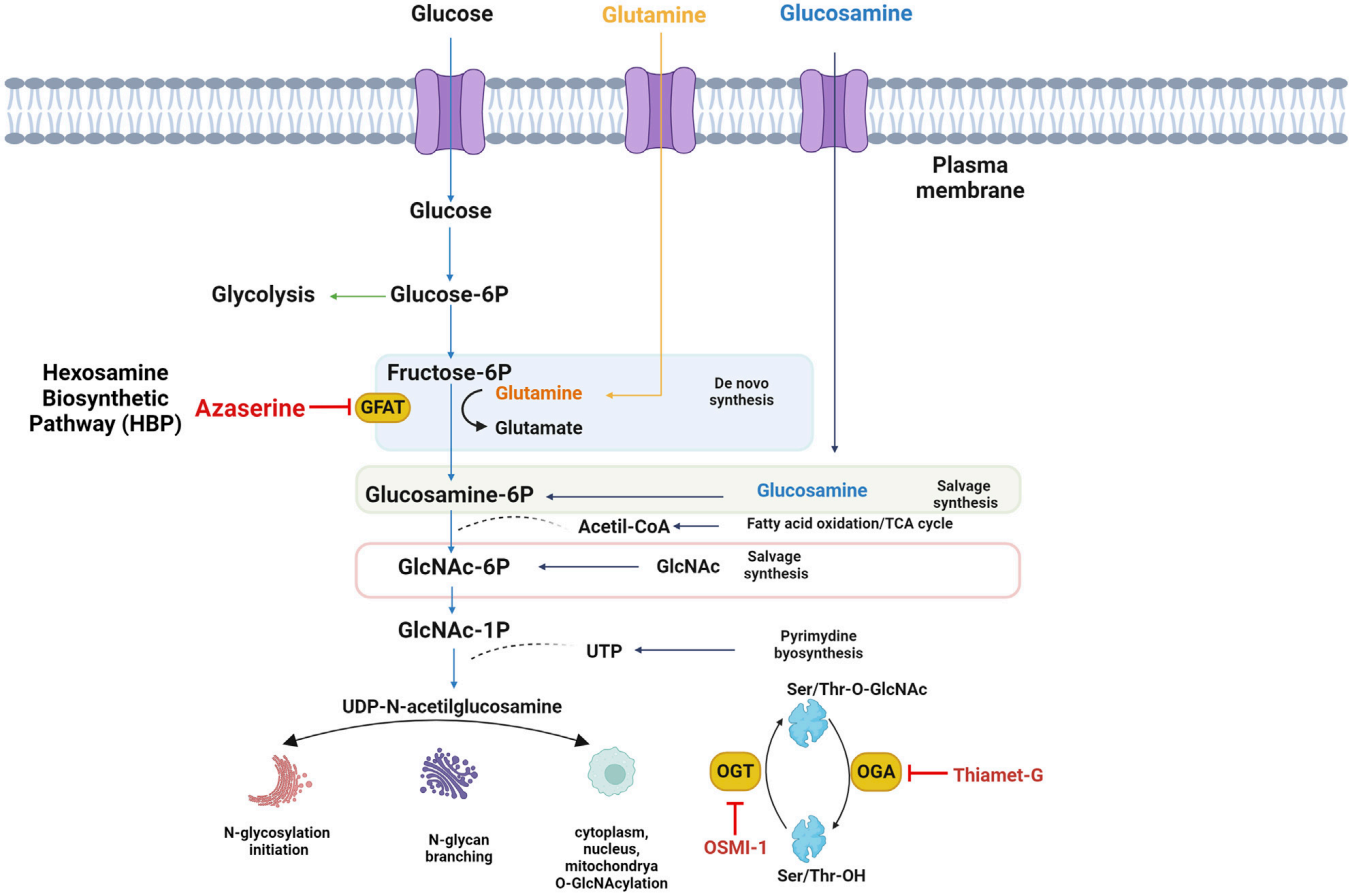
The Hexosamine Biosynthetic Pathway (HBP) and O-GlcNAcylation. Fed for glucose and glucosamine, UDP-GlcNAc, the final product of HBP, which is involved in the synthesis of glycoproteins and proteoglycans, integrates multiple metabolic pathways and has long been considered an important nutrient signaling pathway, L-glutamine: D-fructose-6-phosphate amidotransferase (GFAT) is the rate-limiting enzyme of HBP, and the regulation of HBP flux via small-molecule inhibitors of GFAT is limited to the glutamine analogs Azaserine, or OSMI-1 which inhibit the O-GlcNAc transferase, responsible for using UDP-GlcNAc to modify proteins with O-GlcNAc. TMG inhibits the hexosaminidase O-GlcNAcase, which removes O-GlcNAc from proteins.

A small portion of UDP-GlcNAc acts as a versatile substrate pool for reversible post-translational modifications known as O-GlcNAc. This process, which occurs in the cytoplasm, mitochondria, and nucleus, shares common features with phosphorylation. O-GlcNAcylation and phosphorylation target serine or threonine residues, leading to a dynamic interplay as they compete for the same or adjacent sites (Leney et al., 2017). Furthermore, O-GlcNAcylation engages in intricate crosstalk with other PTMs, such as acetylation, methylation, ubiquitination, and proteolysis, offering a myriad of combinations that give rise to diverse protein isoforms in the human proteome (Leutert et al., 2021). Moreover, protein O-GlcNAcylation is very sensitive to the availability of uridine di-phospho-N-acetylglucosamine, the GlcNAc donor for O-GlcNAcylation, and a downstream metabolite of glucose; therefore, O-GlcNAcylation is often referred to as a nutrient sensor in cells (Hardivillé and Hart, 2014). A single N-acetylglucosamine group is attached to the serine and threonine residues of target proteins by O-GlcNAc transferase. In contrast, the enzyme O-GlcNAcase manages the removal of this group (Fisi et al., 2017) (Figure 1). OGT interacts with several intracellular proteins and O-GlcNAcylation to modify proteins associated with various human diseases such as cancer, neurodegenerative and cardiovascular diseases (Nie and Yi, 2019). Rescue metabolites such as glucosamine and N-acetylglucosamine are used to modulate flux through the hexosamine pathway, and pharmacological agents or anti-metabolites have been used to alter activity and/or substrate for research purposes or therapeutic benefit. Since GFAT1 catalyzes the main step in the *de novo* synthesis of HBP, many studies have used inhibitors of this enzyme, which are mainly glutamine analogs as cytotoxic antimetabolites of glutamine, Azaserine is used to study glutamine-dependent metabolic pathways (Van Cura et al., 2023). OSMI-1 has been identified as a permeable Hogt inhibitor. OSMI-1 binds to the active site of hOGT and inhibits the enzyme with an IC50 value of 2.7 µM *in vitro* (Ju Kim, 2020). On the other hand, Thiamet G is a stable sugar-based inhibitor of the OGA enzyme with excellent inhibitory efficacy (Ki = 21 nM) and good selectivity over human lysosomal β-hexosaminidase (Liu et al., 2017). The effect of glycosylations on the CD36 protein is unclear, but our study shows preliminary results on the localization of CD36 in response to UDP-GlcNAc levels, using HBP pathway and O-GlcNAcylation modulators.

## Materials and methods

### Cell culture

The J774.2 mouse bone marrow-derived macrophage cell line was maintained. In RPMI-1640 medium supplemented with 10% fetal bovine serum (FBS) and 1% antibiotic penicillin-streptomycin at 37°C in 5% CO2.

### Western blot

J774 macrophages were cultured in 6-well plates (9.6 cm^2^), at 1.2 × 10^6^ cells to confluence for 1, 8, and 12 h in RPMI 1640 medium supplemented with 1% FBS, in the presence or absence of 10 µM of TMG (SML0244 Sigma), 50 µM OSMI-1 (SML1621 Sigma) or 0.5 µM Azaserine (A4142 Sigma). The concentrations of the HBP modulators were taken from the following references: OSMI-1 (Ortiz-Meoz et al., 2015), Azaserine (Rajapakse et al., 2009), and TMG (Wani et al., 2017). Cells were then washed with ice-cold PBS and lysed with buffer 50 mM Tris–HCl (pH 8), 137 mM NaCl, 10% (v/v) glycerol, 1% (v/v) Triton, 50 mM NaF, 10 mM disodium β-glycerophosphate, 1 mM Na_3_VO_4_, and protease inhibitor cocktail tablets (ROCHE) supplemented with 100 µM PUGNAc (A7229 Sigma) and 4 µM TMG (SML0244 Sigma), to preserve the O-GlcNAcylation state of the proteins during the extraction procedure. The quantification of protein content was determined using the BCA Protein Assay Kit (Thermo Scientific™ 23225), according to the manufacturer’s instructions. Forty µg of total protein was resolved by a 10% reduction SDS-PAGE using Tris-Glycine gels and transferred to nitrocellulose membranes (GE Healthcare) at constant amperage (10 V for 14 h). The transfer efficiency and equal loading were verified using Ponceau red staining (17435103 Thermo Scientific). Membranes were first saturated for 60 min with 5% (m/v) nonfat dry milk in Tris-buffered saline (TBS)- Tween buffer [15 mM Tris·HCl, 140 mM NaCl, and 0.05% Tween 20 (vol/vol), pH 8.0]. We used mouse monoclonal anti-O-GlcNAc (RL2, ab2739) at a dilution of 1:1,000, and rabbit polyclonal anti-β-actin (4,967 Cell Signaling) at a dilution of 1:1,000 was used as a loading control. After incubation with appropriate antibodies, the bands were visualized using enhanced chemiluminescence reagents (ECL Select, Amersham) and detected with a chemiluminescence ImageQuant™ 500.

### Signal density quantification by ImageJ

All western blots were performed independently in triplicate. The obtained images were analyzed by ImageJ software. First, they were converted to 8-bit format, then each “O-GlcNAc protein profile was individually selected and circumscribed with the rectangular ROI selection and “gels” function, followed by quantification of the peak area of the obtained histograms. For more details on the configuration of “gel analyzer options,” see supplementary materials. Data were acquired as arbitrary area values, and the differences in the expression between groups were normalized to β-actin. All data are presented as the means ± standard deviation for all performed repetitions. Statistically significant differences among three or more groups were analyzed by one-way analysis of variance, followed by Tukey’s *post hoc* analysis. *, **, ***, *p* < 0.05, *p* < 0.001, *p* < 0.0001, respectively, ns non-significant.

### Immunofluorescence

J774 cells were grown on a Chamber Slide System (Thermo Fisher Scientific) for 12 h in 1% FBS in the absence or presence of TMG [10 µM], OSMI-1 [50 µM], or Azaserine [0.5 µM]. Cells were washed with Phosphate-buffered saline (PBS), fixed with 4% cold methanol for 20 min, and then washed with PBS. Non-specific binding sites were blocked with IgG-free 2% bovine serum albumin (BSA, Thermo Fisher Scientific) for 30 min at room temperature. Slides were washed and incubated with primary antibody (anti-CD36) diluted 1:200 in PBS with 1% albumin overnight at 4°C in a humidity chamber. Slides were washed with TBS and incubated with a second primary antibody (anti-O-GlcNAc) diluted 1:100, and Rab-5 (E6N8S Cell Signaling) diluted 1:100, for 90 min in a dark humidity chamber. Then, the chambers were washed with PBS 3 times for 5 min. We incubated the secondary antibodies (Alexa 488-conjugated anti-mouse and Alexa 594-conjugated anti-rabbit) diluted 1:100 at room temperature for 60 min in a dark humidity chamber. Slides were washed, stained, and sealed with Fluoroshield with DAPI-mounting medium (Sigma-Aldrich) and coverslipped. Images were obtained with a Cytation 5 imaging reader.

### Signal fluorescence quantification by ImageJ

First, images were converted to 16 bits and freehand selected to draw on the circumference of the cells; 10 cells were randomly selected per image in 4 independent experiments. The “measure” function was used to obtain an average fluorescence intensity. Statistically significant differences between groups were analyzed by one-way analysis of variance, followed by Tukey’s *post hoc* analysis. *, **, ***, *p* < 0.05, *p* < 0.001, *p* < 0.0001, respectively, ns non-significant.

### Signal fluorescence colocalization by ImageJ

Images were converted to 8-bit and analyzed with the JACoP plug-in of the ImageJ software (2006.02.01). The JACoP colocalization plug-in primarily uses statistics to assess the relationship between fluorescence intensities (Bolte and Cordelières, 2006), using Pearson correlation coefficients that measure the strength of the linear relationship between two variables, the gray values of fluorescence intensity pixels of green and red image pairs, this is mostly done using correlation coefficients that measure the strength of the linear relationship between two variables, in this case the gray values of fluorescence intensity pixels of green and red image pairs. Subsequently, statistically significant differences between groups were analyzed by one-way analysis of variance, followed by Tukey’s *post hoc* analysis. *, **, ***, *p* < 0.05, *p* < 0.001, *p* < 0.0001, respectively, ns non-significant.

## Results

### O-GlcNAcylation protein levels respond according to the time treatment with modulators

**FIGURE 2:**
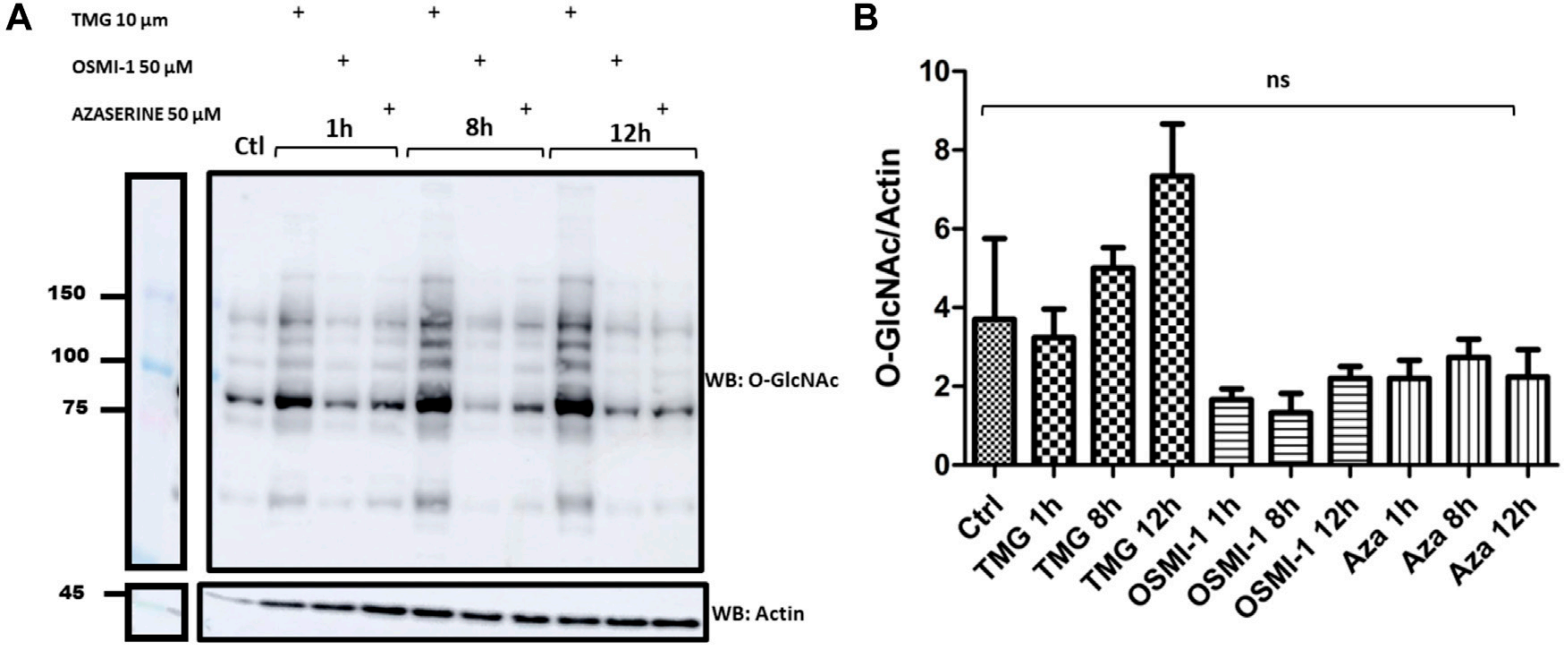
Western Blots of the profile of O-GlcNAcylated proteins in J774 macrophages. **(A)** O-GlcNAc profile in cells without stimulation (control), in the presence or absence of TMG (10 µM), OSMI-1 (50 µM), or Azaserine (0.5 µM), during 1, 8 and 12 h. We incubated Western Blots with anti-O-GlcNAc and anti-actin (as a loading control). **(B)** The effect of TMG, OSMI-1, and Azaserine treatment on O-GlcNAc/actin protein expression is observed. Graphs represent the means ±3 independent experiments. Statistical analysis was performed using a one-way analysis of variance followed by Tukey’s *post hoc* test. *, **, ***, *p* < 0.05, *p* < 0.001, *p* < 0.0001, respectively, ns non-significant.

To analyze the effect of TMG, OSMI-1, and Azaserine on the levels of O-GlcNAcylated proteins, we treated J774 macrophages in the presence of 10 µM TMG as the optimal dose, 50 µM OSMI-1, or 0.5 µm Azaserine, the signal detected in the blot was measured by densitometry (Figure 2A), shows O-GlcNAc profile proteins in response to treatments; we noticed that when J774 cells were treated with TMG at 8 and 12 h, they had a tendency to exhibit more O-GlcNAcylation compared to the control. OSMI-1 and Azaserine did not show variations between treatment times, otherwise a better response was observed at 12 h of TMG treatment; after these results, we decided to perform the next experiments at 12 h.

### CD36 protein colocalization with O-GlcNAc and Rab-5

To explore the effects of TMG, OSMI-1, and Azaserine treatment on the cellular distribution of CD36, J774 cells were examined using immunofluorescence, and we evaluated the colocalization using Pearson correlation with the JACoP plug-in of the ImageJ software. In control cells, CD36 and Rab-5 show a large distribution in intracellular compartments. The increase in UDP-GlcNAc availability under TMG treatment shows a wide distribution to intracellular compartments and areas with increased staining of CD36, which colocalizes with Rab-5, located near the plasma membrane (yellow arrows). Treatment with the OSMI-1 and Azaserine generated a redistribution of CD36 from the cytoplasm to the surface (white arrows). CD36 and Rab-5 colocalization showed a significant decrease in Pearson correlation after treatment with OSMI-1 and Azaserine (*p* < 0.05) (see supplementary materials). These results suggest that CD36 trafficking is sensitive to the environmental status of UDP-GlcNAc.

## Discussion

To evaluate the effect of modulators TMG, OSMI-1, and Azaserine on O-GlcNAc protein levels, we treated J774 macrophages. The effects of increased/decreased O-GlcNAcylation levels, depending on the treatment with the aforementioned modulators, were evaluated using an anti-O-GlcNAc antibody (Rl2). The treatment with OSMI-1 and Azaserine demonstrated a non-significant reduction of O-GlcNAcylated proteins, because their formation depends on the availability of UDP-GlcNAc as a substrate (Rajapakse et al., 2009; Olivier-Van Stichelen et al., 2012; Chiaradonna et al., 2018).

**FIGURE 3:**
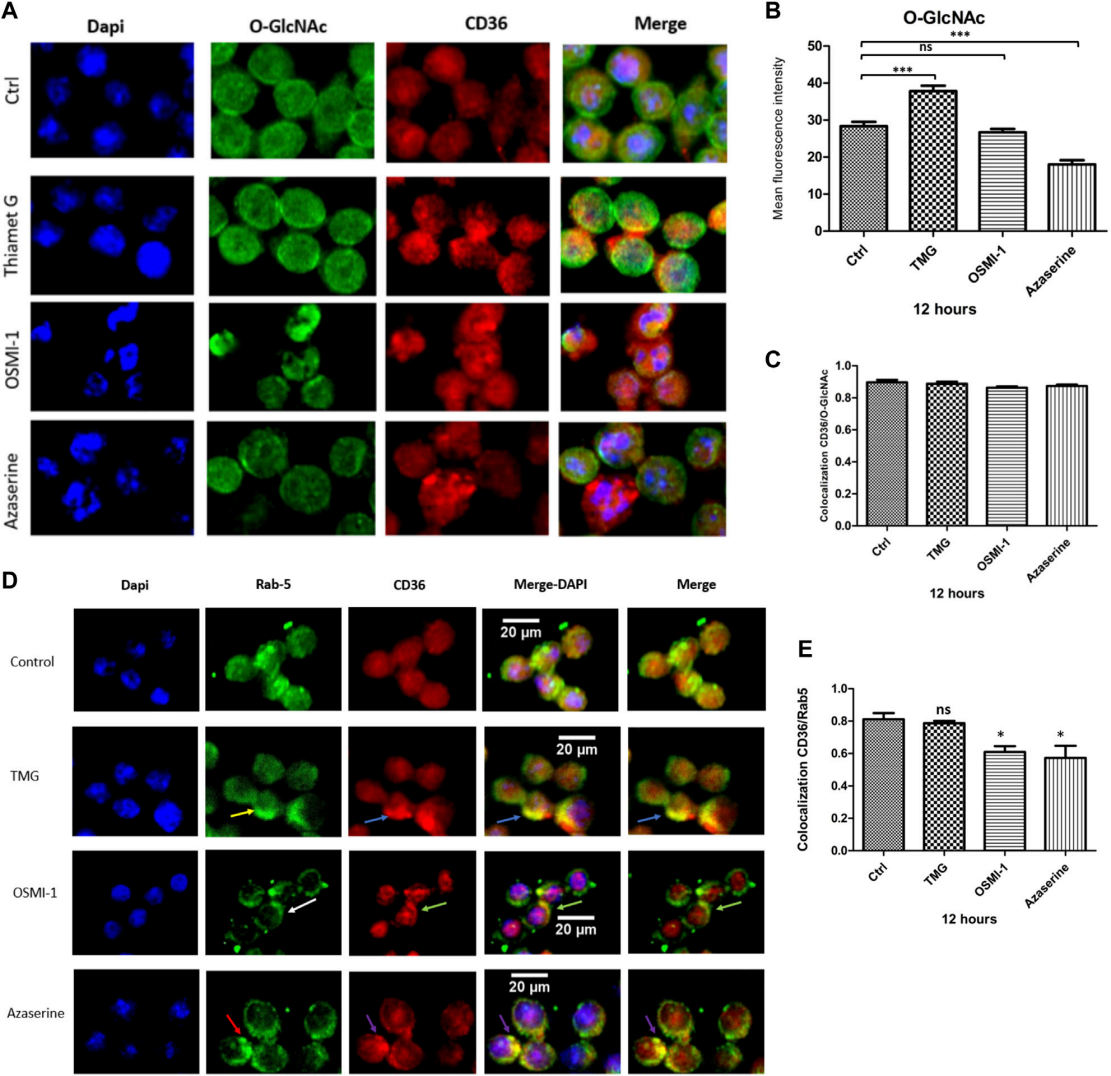
CD36 protein colocalization with O-GlcNAc and Rab-5. Macrophages were cultured with RPMI medium either in the presence or absence of TMG, OSMI-1, or Azaserine for 12 h and processed for immunofluorescence, as described in the materials and methods section. **(A)** Representative images of immunofluorescence staining for O-GlcNAc (green), CD36 (red), and merged with cell nuclei (blue). **(B)** Histograms represent the means ± SE from four independent experiments of mean fluorescence intensity of O-GlcNAcylation levels (green), with different treatments of TMG, OSMI-1, and Azaserine for 12 h. **(C)** The graph represents the means ± SE from four independent experiments to measure the Pearson correlation of CD36 and O-GlcNAc colocalization, using the JACoP plug-in of the ImageJ software. **(D)** Representative images of immunofluorescence staining for cell nuclei (blue), Rab-5 (green), and CD36 (red), merged with DAPI and Rab-5/CD36. **(E)** Bar graphs represent the means ± SE from four independent experiments of Pearson correlation of Rab-5/CD36 colocalization using the JACoP plug-in in ImageJ. * Significant as compared with untreated controls (*p* < 0.05), using ANOVA followed by Tukey’s *post hoc* test, *, **, ***, *p* < 0.05, *p* < 0.001, *p* < 0.0001, respectively, ns; non-.significant.

An increase in global O-GlcNAcylation levels was observed when J774 cells were treated with TMG, for 8 and 12 h (Figure 2). Immunofluorescence staining showed significant changes in the mean fluorescence intensity of O-GlcNAcylation after Azaserine treatment (*p* < 0.0001), and we observed a tendency to decrease O-GlcNAcylation after treatment with OSMI-1. We significantly increased O-GlcNAcylation levels by applying TMG treatment (*p* < 0.0001) (Figures 3A, B). These results confirm the function of the modulators. CD36 and O-GlcNAc showed high correlation rates with the control, and we detected no changes between the treatments compared to the control (Figure 3C).

Subsequently, we evaluated whether the availability of UDP-GlcNAc could impact the localization of CD36. We assessed the localization of CD36 by immunofluorescence; J774 macrophages were treated with TMG, OSMI-1, and Azaserine for 12 h. Under basal conditions, a wide and even distribution of CD36 was observed, both in the cytoplasm and in the plasma membrane. Proteins bound to the Rab-5 antibody were observed predominantly close to the plasma membrane (yellow arrows) and still present in the cytoplasm, probably in intracellular vesicles. When macrophages were treated with TMG, CD36 was observed to be increased in specific areas close to the plasma membrane (blue arrows), although we obtained non-significant changes compared to control, in colocalization results. On the other hand, after OSMI-1 treatment, CD36 and Rab-5 tended to redistribute close to the plasma membrane (white and green arrows, respectively). Treatment with Azaserine also redistributed CD36 (red arrows) and Rab-5 (purple arrows) to the plasma membrane (Figure 3D). The colocalization between CD36 and Rab-5 showed a significant decrease with OSMI-1 and Azaserine treatment (Figure 3E).

One study suggested that the N-glycosylations present in CD36 are responsible for the increase of CD36 to the membrane and the β-oxidation of oleate in rat hearts after treatment with N-acetylglucosamine (feeder of the hexosamine pathway), which would therefore increase the levels of UDP-GlcNAc favoring the levels of N-glycosylation (Lauzier et al., 2011). Alternatively, another modification involved in the recruitment of CD36 to the plasma membrane may be O-GlcNAc, as demonstrated by Laczy, when perfusing mouse hearts with N-acetylglucosamine, where the binding of CD36 to OGT was increased, improving fatty acid absorption (Laczy et al., 2011). Our results suggest that the O-GlcNAc modification may play a role in the recruitment of CD36 to the macrophage membrane following treatment with TMG. TMG inhibits the OGA enzyme, thereby increasing O-GlcNAcylation levels. After this treatment, CD36 is predominantly found near the plasma membrane predominantly, after this treatment.

There is evidence that both N-glycosylation and O-GlcNAc modification can contribute to the regulation of membrane trafficking. These modifications depend on the concentration of UDP-GlcNAc, generated by the HBP, through the intervention of several metabolites, such as glucose, glutamine, ATP, and Acetyl-CoA, depending on the environmental conditions, which are increasingly considered central regulators that affect cellular physiology (Bond and Hanover, 2015). The control of membrane trafficking can result from the direct modification of the receptor with O-GlcNAc or through the O-GlcNAcylation of other proteins that regulate endocytic trafficking processes, for example, modifying coat proteins involved in membrane fusion and fission (Guo et al., 2014), or proteins involved in clathrin-mediated endocytosis (Guo et al., 2014; Rahmani et al., 2019).

The decrease in colocalization suggests a redistribution of CD36 close to the plasma membrane after OSMI-1 and Azaserine treatment. This evidence also suggests that CD36 localization may be influenced by the nutrient availability of the cell, which determines the level of UDP-GlcNAc through the flux of the HBP. As mentioned, there are many examples that demonstrate the regulation of membrane trafficking through post-translational modifications; however, much remains to be discovered about the details of these mechanisms. One study that evaluated the impact of O-GlcNAc levels on cellular metabolism and energy homeostasis involved AMPK, a heterotrimeric complex signaling protein with many targets that regulate a wide range of cellular processes. AMPK upregulates catabolism, inactivates anabolism, and modulates endocytic traffic (Li and Chen, 2019). AMPK phosphorylation increases and ameliorates autophagic flux after inhibiting O-GlcNAcylation. Jin L et al. suggest that O-GlcNAcylation behaves like a regulator of AMPK activity (Jin et al., 2020).

Pathogen recognition, antigen presentation, and macrophage homeostasis are mediated by various receptors, including mannose and scavenger receptors. It has been reported that these receptors co-localize with Rab5 (Mukherjee et al., 2002), and IL-4/PGE2 stimulation significantly increases the expression of the mannose receptor, Rab5, and the Rab5 GEF Rin in mouse bone marrow-derived macrophages (Wainszelbaum et al., 2006). The recycling of CD36 from endosomes to the plasma membrane and *vice versa* is regulated by different families of proteins, adapter proteins (Scales et al., 1999), and Rab-GTPases, which regulate the binding of coat proteins to CD36-enriched areas, leading to the generation of a vesicle that moves through the filaments of the cytoskeleton to its target membrane (Glatz and Luiken, 2018). The Ras superfamily of GTPases is extensively modified by posttranslational modifications like prenylation, phosphorylation, and ubiquitination (Shinde and Maddika, 2018), which facilitate their membrane attachment and determine their subcellular localization and function; phosphorylation could trap the GDP-bound Rab either in the cytosol or at the membrane (Konstantinopoulos et al., 2007). In Hep3B cells, hyper O-GlcNAc modification regulates Rab3A, which attenuates the tumor suppressor effect of Rab3A on hepatocellular carcinoma (HCC) metastasis (Wu et al., 2018). Rab11, another traffic membrane regulator, is also modified by O-GlcNAc (Zhong et al., 2015). The evidence above suggests that glycosylations influence vesicular traffic by directly modifying membrane receptors like CD36 and impacting proteins involved in vesicular traffic, like Rab proteins.

The structure and function of receptors and vesicular trafficking regulatory proteins are mediated by modifications such as glycosylation, that are sensitive to metabolic conditions and impact different physiological contexts, health, and disease. The complexity of glycosylation on CD36 needs to be supported by further research. The structural variation presented by N-glycans is regulated by the modulation in the expression profile and activity of glycosyltransferases and glycosidases, leading to specific N-glycosylation structures in each cell type (Croset et al., 2012; Butler and Spearman, 2014). Direct glycosylation or phosphorylation of glycosyltransferases promotes their activity (Agrawal et al., 2014). Furthermore, glycosyltransferases and glycosidases and/or the availability of their specific substrates also modulate the glycosylation profile (Paneque et al., 2023). Then, the modulation of N-glycosylation is regulated at the transcriptional and post-translational levels and by the availability of substrates, revealing the complexity of regulating N-glycosylation (Esmail and Manolson, 2021). Nutrient availability regulates cellular O-GlcNAcylation levels by determining the amount of UDP-GlcNAc and modulating the levels of OGT, OGA, and their respective adapter proteins and substrates. Similarly, the activity of the OGT enzyme is regulated by the nutritional conditions of the cell. In response to systemic changes in the metabolic state (through hormonal signals) (Yang and Qian, 2017), specific adapter proteins intervene to O-GlcNAcylate a specific substrate, leading to the activation of key cellular pathways, such as suppression of anabolism, stimulation of gluconeogenesis and ketogenesis, and activation of thermogenesis (Yang and Qian, 2017). This study modified the availability of a single substrate, UDP-GlcNAc, a variable within a complex network that regulates glycosylations. Further investigation using CD36 mutants at specific glycosylation sites may provide more compelling results for understanding its biological effects in folding, maturation, trafficking, secretion, and function.

These results are intended to set a pattern to continue discovering the impact of glycosylations on macrophage functions related to CD36. There are some questions to be addressed, such as whether aberrant modification of these glycosylations could uniquely influence localization, similarly influence inflammatory functions and lipid uptake, or disrupt the physiological conditions of macrophages. Our results suggest that UDP-GlcNAc is available for influx into CD36 and Rab-5 redistribution between the cytoplasm and cell surface.

## Limitations of the study

1. Dependence on Specific Modulators: The conclusions of this study rely heavily on the effects of specific chemical modulators (Thiamet G, OSMI-1, and Azaserine) on the HBP. The observed responses may be specific to these compounds and may not fully represent the natural physiological regulation of O-GlcNAcylation. Hence, gene manipulations of OGT, OGA, GFAT, and CD36, will be required in addition to the use of agonists/antagonists of CD36.
2. *In vitro* Model: While the J774 macrophage cell line is a valuable tool, it limits the applicability of the findings to *in vivo* systems, where the cellular environment and interactions are more complex. The results obtained *in vitro* may not fully translate to *in vivo* contexts due to differences in the cellular microenvironment and systemic factors, underscoring the need for further research in this area.
3. Single Time-Point Analysis: Although necessary, the study’s focus on specific time points (e.g., 12 h post-treatment) to assess the effects of modulation on protein O-GlcNAcylation and localization may miss transient changes and dynamic processes at other time intervals. A more comprehensive time-point analysis could provide a more nuanced understanding of these processes.
4. Limitations of Quantitative Analysis: The methods used to quantify O-GlcNAcylation levels and protein localization, such as densitometry of Western blots and fluorescence intensity measurements in immunofluorescence, have inherent limitations in precision and reproducibility. These methods are heavily dependent on the linearity and sensitivity of the detection systems and may not accurately reflect subtle changes in O-GlcNAcylation or protein distribution. Other loading controls, such as β-tubulin or clathrin, may be considered in future studies.
5. Lack of Direct Evidence for Mechanistic Pathways: While the study discusses the potential mechanisms by which O-GlcNAcylation affects protein function and localization, it needs more direct experimental evidence linking specific O-GlcNAcylation changes to observed functional outcomes. Molecular studies or genetic interventions that directly alter O-GlcNAcylation sites on CD36 may provide more conclusive evidence of causality.
6. Generalizability of Findings: The findings are derived from a single type of immune cell (macrophages) and may not be generalizable to other cell types involved in the immune response, such as T cells, B cells, or dendritic cells, which may have different O-GlcNAcylation dynamics and functional responses.

## Data Availability

The original contributions presented in the study are included in the article/supplementary material, further inquiries can be directed to the corresponding author.
